# Cyanotoxin release from the benthic, mat-forming cyanobacterium *Microseira* (*Lyngbya) wollei* in the St. Lawrence River, Canada

**DOI:** 10.1007/s11356-020-09290-2

**Published:** 2020-05-26

**Authors:** Sylvie Poirier-Larabie, Christiane Hudon, Hugo-Pierre Poirier Richard, Christian Gagnon

**Affiliations:** Environment and Climate Change Canada, 105 McGill, Montréal, Québec H2Y 2E7 Canada

**Keywords:** Cyanotoxin release, *Microseira (Lyngbya) wollei*, Mass spectrometry, Benthic cyanobacterial mat

## Abstract

Benthic cyanobacterial mats occurring in the St. Lawrence River fluvial lakes Saint-Louis and Saint-Pierre are dominated by *Microseira (Lyngbya) wollei* which produce several cyanotoxins including LWTX-1 that is characteristic of *Microseira wollei*. This cyanotoxin is not only present in the filaments forming benthic mats, but was also measured in the water overlying the mats. LWTX-1 was found in all cyanobacterial filament samples (75.29–103.26 ng mg^−1^) and all overlying water samples (3.01–11.03 ng L^−1^). Toxin concentrations measured in overlying water and dry biomass were strongly correlated (*r* = 0.94). Furthermore, LWTX-1 concentration in water was positively correlated with the dissolved organic carbon in water (*r* = 0.74) and % nitrogen content in cyanobacterial filaments (*r* = 0.52). A preliminary study was conducted to determine the release and degradation rates of LWTX-1 from a *M. wollei* mat kept under laboratory conditions over a 3-month period. Toxin measurements revealed an early, massive toxin release followed by a typical decaying function, with a half-life in the order of 17 days. Our results raise concerns about the occurrence and downstream advection of dissolved cyanotoxins from *Microseira* mats in the aquatic environment.

**Figure:**
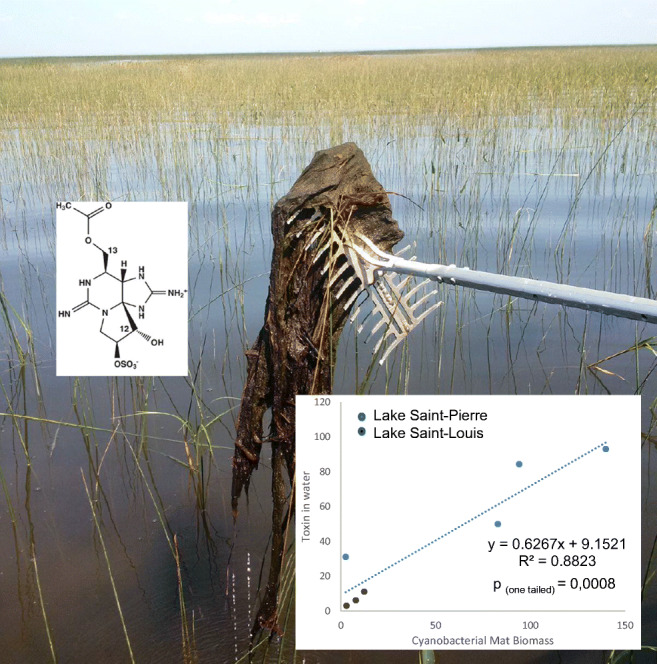
Graphical abstract

Graphical abstract

## Introduction

Harmful algal blooms (HABs) of benthic cyanobacteria mats of *Microseira* (formerly *Lyngbya) wollei* (Farlow ex Gomont) G.B.McGregor & Sendall ex Kenins (Guiry and Guiry 2014; Kenins 2017) have been observed for several years in freshwaters around the globe (Onodera et al. 1997; Foss et al. 2012; Hudon et al. 2016; Smith et al. 2019). Causes of proliferation include eutrophication resulting from human activities, warming water temperatures, global warming (Paerl and Huisman 2009; O’Neil et al. 2012; Paerl and Paul 2012; Paerl et al. 2016; Visser et al. 2016), and qualitative and quantitative load of nutrients near watersheds and airsheds (O’Neil et al. 2012; Paerl et al. 2016). Cyanotoxins from benthic cyanobacteria mats of *Microseira wollei* have been characterized for molecular structures and environmental concentrations (Onodera et al. 1997; Seifert et al. 2007; Foss et al. 2012; Gaget et al. 2017; D’Agostino et al. 2019). They contain, among others, cyanotoxins of the paralytic shellfish poison (PSP) family as saxitoxin analogs (STX) such as mainly decarbamoylgonyautoxins 2 & 3 (dcGTX 2 & 3) and decarbamoylsaxitoxin (dcSTX), some other analogs, and *microseira*-specific LWTX 1–6 (Paerl and Huisman 2009). It may also produce cylindrospermopsin (CYN), deoxycylindrospermopsin (deoCYN), and some microcystins (O’Neil et al. 2012; Paerl et al. 2016). The toxicity of these cyanotoxins can vary widely. Indeed, as demonstrated by Onodera et al. (1997) and by Oshima (1995), the toxicity of STX, dcSTX, and dcGTX 2 & 3 is much higher (1617–2483 mouse unit (MU, μmol^−1^)) than that of LWTX 1–6 (˂ 10–326 MU (μmol^−1^)). Also, these cyanotoxins demonstrate different types of toxicity such as neurotoxicity (saxitoxin), dermatotoxicity (LWTX-1), hepatotoxicity, and cytotoxicity (cylindrospermopsin) (Onodera et al. 1997; Roy-Lachapelle 2016).

For planktonic and benthic (including *Microseira)* cyanobacteria, the environmental factors controlling cyanotoxin production and release into the environment are still poorly unknown (Sivonen and Jones 1999). Planktonic cyanobacteria appear to produce the highest toxin concentrations under the light, temperature, and nutrient concentrations most favorable for their growth. In culture, the rate of microcystin production was directly proportional to population growth rate, no matter what environmental factor was limiting growth (Orr and Jones 1998): toxin concentrations rose during the period of active cell growth, reaching a maximum during or in the late logarithmic phase (Sivonen 1990). Cellular decay, death, and lysis of planktonic cyanobacteria lead to toxin release in the environment (Chorus and Bartram 1999), potentially leading to toxin assimilation by benthic macroinvertebrates (Umehara et al. 2017). As with planktonic cyanobacteria, toxin concentrations in *Microseira (Lyngbya) wollei* were positively correlated with in situ biomass and possibly to the physiological status of filaments (Hudon et al. 2016), although the mechanisms triggering toxin release in benthic cyanobacterial mats remain to be ascertained.

The large level of spatial (between and within fluvial lakes) and temporal (seasonal, inter-annual 2006–2013) variability in *Microseira wollei* biomass and LWTX-1 concentration in mats has been previously demonstrated in the St. Lawrence River (Hudon et al. 2016), raising the hypothesis that *Microseira wollei* could release cyanotoxins into the overlying waters in proportion with the biomass of benthic mats of filaments. As an insight, the wash water from cleaning up the sampled filament mats from detritus and sediment contained a thousand times more LWTX-1 (result not shown) than measured in samples from the water column. For this reason, it was hypothesized that even if the cyanobacterial mats were healthy, and not about to die and release its toxins by cell lysis, any disturbance of mats (such related to episodes of wind, waves, or current) could stimulate the release of toxins. The main mechanism of toxin release from *Microseira wollei* cells is unknown so far. There is some evidence that not only cell lysis is responsible for algal toxin release. Walls et al. (2018) demonstrated with microcystin sp. (both in laboratory and in situ) that temperature regulates the production of harmful cyanobacteria and the release of toxins. Several combinations of light and temperature conditions have shown impact on growth rate and influence on the production and release of cylindrospermopsin (Preußel et al. 2009).

**Fig. 1:**
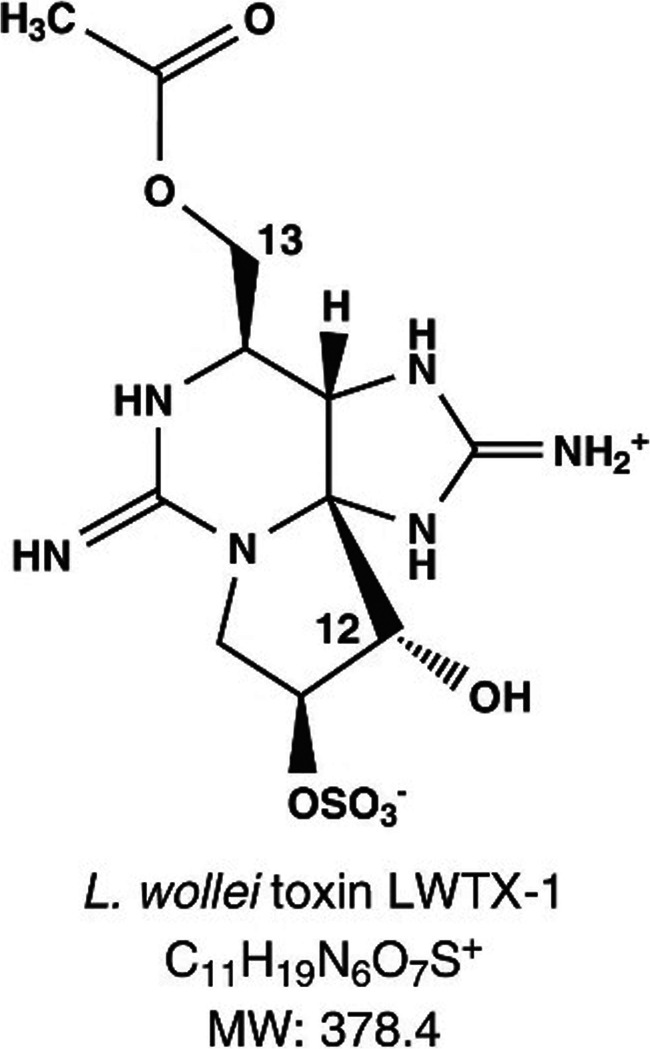
Molecular structure of LWTX-1 toxin

LWTX-1 toxin (Fig. 1) is characteristic of the benthic cyanobacterial mats of *Microseira wollei* which predominate in the St. Lawrence River fluvial lakes (Lajeunesse et al. 2012; D’Agostino et al. 2019). This relatively mild toxin is not found in pelagic cyanobacteria (Onodera et al. 1997). It is therefore a good tracer of the benthic cyanobacteria mats of *Microseira wollei* and the release of this toxin in the water column could indicate its presence as well as the presence of the other associated cyanotoxins of this benthic algae (D’Agostino et al. 2019). Since a reference standard was available for this toxin, albeit not certified at the beginning of this study but became certified in 2018 by National Research Council Canada (NRC), LWTX-1 was therefore selected in order to study the release of toxins from algae mats in Lake Saint-Louis and Saint-Pierre.

**Fig. 2:**
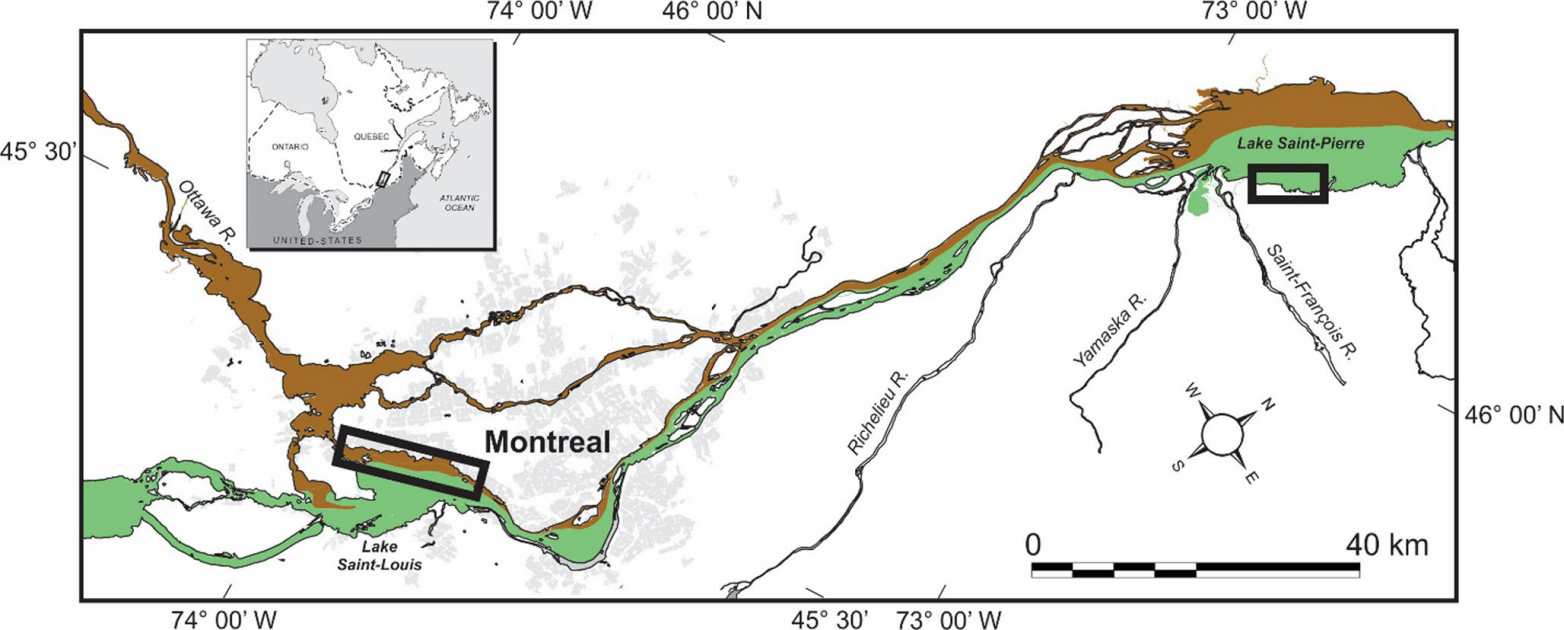
Location of *M. wollei* sampling areas in fluvial lakes Saint-Louis and Saint-Pierre (rectangular boxes), St. Lawrence River (QC, Canada, see inset). Urbanized areas (in gray) and major tributaries are shown: waters from Great Lakes (green) and Ottawa River (brown)

Subsequently, the LWTX-1 toxin release rate and decay from a cyanobacterial mat kept under stable laboratory conditions were evaluated in order to estimate its persistence in the environment. The fate of some cyanobacterial metabolites released in water was reported (microcystins, nodularin, cylindrospermopsin) (Hiskia et al. 2014) but there is still a lack of data regarding the fate of LWTX-1. Detection and quantification of LWTX-1 released by cyanobacterial mats in the overlying water required the development of an extraction and purification method to reduce the matrix effect while concentrating the toxin to offset its dilution by river waters. The accurate measurement of released toxins requires the use of adequate analytical blanks, that are, waters unaffected by cyanobacteria, yet bearing a chemical signature similar enough to correct for the matrix effect. This challenge is particularly important in the St. Lawrence River, where *Microseira wollei* rarely occurs in waters originating from Lake Ontario but rather proliferates in shorewaters influenced by brown, nutrient-rich tributaries draining farmlands (Lévesque et al. 2017a). In Lake Saint-Louis, *M. wollei* is exclusively found in the Ottawa River waters flowing along the north shore, whereas in Lake Saint-Pierre, cyanobacterial mats are concentrated along the south shore, in waters influenced by the Yamaska and St. François rivers (Fig. 2). The choices of matrix blanks were therefore made considering the presence or absence of LWTX-1 and the physicochemical characteristics of the tributary upstream of the sampling site.

## Material and methods

### Material and standards

Standard of cyanotoxins (LWTX-1) (11 μM in 17 μM acetic acid) (not certified) was obtained from the Marine Analytical Chemistry Standard Program (NRC, Halifax, NS, Canada). Diluted LWTX-1 calibration solutions were prepared in acetic acid (0.01 M) and stored at − 20 °C in amber glass bottles. LC-MS grade water (H_2_O), methanol (MeOH), and acetonitrile (ACN) were purchased from Fisher Scientific Canada (Ottawa, ON). Ammonium bicarbonate (NH_4_HCO_3_), acetic acid (CH_3_COOH), ammonium formate (NH_4_HCO_2_), formic acid (HCOOH), and ammonium hydroxide (NH_4_OH) were purchased from Sigma-Aldrich Canada (Oakville, ON). Deionized water (DI-H_2_O) passed through a Milli-Q Advantage A10 system (Millipore, Billerica, MA) was used for method blanks. This system is equipped with activated carbon, an ion exchange resin, and a UV lamp to reduce total organic carbon (TOC) to ≤ 5 ppb and increase resistivity (≥ 18.0 MΩ cm). Tap water treated with activated charcoal filter, 5-μm particulate filter, and UV irradiation unit was used for the degradation of biomass mat in aquarium.

### Sample collection and preservation and characterization

#### Study sites

Samples were collected in the shallow (< 3 m), slow flowing (< 40 cm s^−1^) littoral areas of fluvial lakes Saint-Louis and Saint-Pierre, which are enlargements (respectively 148 and 300 km2) of the St. Lawrence River (Fig. 2). Lake Saint-Louis is located at the confluence with the Ottawa River, in the largely urbanized Montreal area. In contrast, Lake Saint-Pierre lies 65 km downstream, in a rural setting. Mean (± SD) annual St. Lawrence River discharge at the outlet of Lake Saint-Louis was markedly higher in 2017 (10,015 ± 1540 m3 s^−1^) than the 2000–2016 average (8090 ± 1013 m3 s^−1^). In both fluvial lakes, waters originating from Lake Ontario flow in the central navigation channel, with little lateral mixing with waters from the Ottawa River (flowing along the north shore) and other tributaries.

#### Field sampling

Water samples and biomass of *Microseira wollei* were collected at the end of the summer in lakes Saint-Louis (15 August 2017, *N* = 4) and Saint-Pierre (28 August 2017, *N* = 3). Water samples for dissolved LWTX-1 measurements and for water quality analyses were pumped 15 cm above the cyanobacterial mat in pre-conditioned 1-L Nalgene polypropylene bottles. Water samples were kept on ice after sampling and subsequently stored at − 20 °C until LWTX-1 analysis. The biomass of *Microseira wollei* was collected using a 35-cm wide double-headed rake dragged over a distance of about 1 m on the bottom (Yin et al. 2000) and was averaged from 3 strokes sampled around the boat.

#### Characterization of water quality, cyanobacterial biomass, and condition

Unfiltered water subsamples were used for analyses of suspended matter (SM), total phosphorus (TP), and total nitrogen (TN). Filtered water samples (Whatman GF/C) were analyzed for total dissolved phosphorus (TDP), NO_2_ -NO_3_, NH_4_^+^, total dissolved nitrogen (TDN), dissolved organic carbon (DOC), and color (Platinum/Cobalt, Pt/Co) using standard methods (Quebec Laboratory of Environment Testing, Montréal 2011). After collection, mats of filamentous cyanobacteria and accompanying vascular macrophytes were rinsed with running water to eliminate sediments, debris, and invertebrates. Cleaned plant material was then sorted, identified microscopically, weighed, and frozen until drying. Biomass of cyanobacterial mats was largely comprised of *Microseira wollei* filaments (> 95%), with the occasional occurrence of (nontoxic) filamentous chlorophytes, epiphytic diatoms, and *Heteroleibleinia*. Wet biomass (WM) of *M. wollei* was converted to dry mass (DM) using a wet:dry conversion factor of 3.4:1 derived from paired measurements of wet and dry mass. Carbon and nitrogen content (as % of DM) of *M. wollei* filaments was determined on ground samples, using an elemental analyzer (Vario MACRO cube CHNS, Elementar, Germany) (Quebec Laboratory of Environment Testing, Montréal 2011); phosphorus content (as % of DM) of filaments was determined following Stainton et al. (1977).

### Development and validation method: toxin analysis

#### LWTX-1 extraction

LWTX-1 was extracted from cyanobacterial filaments using previously developed methodology (Lajeunesse et al. 2012). Extraction of LWTX-1 from water was carried out using the solid phase extraction (SPE) method, adapted from Onodera et al. and Zervou et al. (2017). Thermo Scientific HyperSep Hypercarb SPE cartridges were tested on a Vac Master Sample Processing Station (International Sorbent Technology®) purchased from Biotage® (Charlotte, NC) for the extraction of the LWTX-1 analyte using 100 mL of river water. These cartridges contain 500 mg of porous graphite carbon sorbent. Cartridges were conditioned by adding 6 mL of MeOH followed by 6 mL 100 mM NH_4_HCO_3_ pH 10. Sample pH was adjusted by adding 10 mL of 100 mM NH_4_HCO_3_ pH 10 to 100 mL of water sample. Sample load flow rate was about 2–4 mL min^−1^. After sample loading, cartridges were washed with 6 mL of MeOH. Analyte was eluted with 2 × 3 mL of MeOH/2% acetic acid. SPE extracts were collected in 10-mL centrifuge tubes and evaporated to dryness under a gentle stream of nitrogen in a dry bath set at 40 °C. Evaporated extracts were then reconstituted to 200 μL with 0.1 M acetic acid, vortexed for 10 s, sonicated for 5 min, and quickly centrifuged and transferred to 2-mL glass vials for LC-QqQMS analysis.

#### Identification and quantification of LWTX-1 using LC-QqQMS

Toxins were analyzed in cyanobacterial filaments and water samples using a 1200 Series liquid chromatographer coupled to an electrospray triple-quadrupole mass spectrometer 6410 (Agilent, Santa Clara, CA). LC-MS/MS parameters are presented in Table 1. LC-MS/MS raw data obtained from LC-QqQMS analysis of water samples were analyzed with the MassHunter quantitative software (Agilent, Santa Clara, CA). Two MRM transitions were selected for each analyte, precursor ion and two fragments ions, the first with higher signal for quantification and the second one for qualitative identification confirmation. The chromatography and mass spectrometry characteristics used were based on previously developed methodology (Lajeunesse et al. 2012). Briefly, the chromatographic separation was performed using an HILIC (hydrophilic interaction liquid chromatography) TSKgel Amide-80 column (3 μm, 2 × 150 mm). The mobile phase was constituted of solvent A: aqueous 5 mM NH_4_OOCH and HCOOH 3.6 mM (pH = 3.5) and solvent B: 95% MeCN-5% aqueous 5 mM NH_4_OOCH and 3.6 mM HCOOH (pH = 3.5). The volume injected was 15 μL.

**Table 1:** MS/MS acquisition parameters

Compound name	Prec ion	Prod ion	Dwell	Frag (V)	CE (V)	Delta EMV	Polarity
LWTX-1	379.1	299.1	200	115	12	500	Positive
LWTX-1	379.1	138.2	200	115	30	500	Positive

*Prec ion* precursor ion; *Prod ion* product ion; *Frag* fragmentation voltage; *CE* collision energy; *Delta EMV* electron multiplication voltage

#### Method validation

Method validation for water extraction was performed by evaluating linearity, precision, accuracy, recovery, and lower limit of quantification. In order to take into account the matrix and its effects on the method, the validation was carried out in the St. Lawrence River surface waters used as a matrix blank. Since the two sampling sites are under the influence of two different water bodies, tributary waters sampled upstream of the areas colonized by *M. wollei* were used as matrix blanks for method development and validation in water. First, St. Lawrence River surface waters originating from Lake Ontario were used as matrix blanks for Lake Saint-Louis samples. Second, Yamaska River surface waters were used as matrix blanks for Lake Saint-Pierre samples. Every validation test was evaluated in those two matrices. Also, since a certified LWTX-1 standard was not available at the beginning of this study, and a noncertified standard was available but in limited quantity, an in-house reference material (RM) was prepared, as additional quality control, from a large batch of field-collected cyanobacterial filaments. A determined amount of freeze-dried, powdered filaments was weighted and mixed with 0.1 M acetic acid prior to heating (100 °C for 5 min). This mixture was quantitatively transferred to an amber bottle and the LWTX-1 concentration was quantified using a (noncertified) standard curve by serial dilutions of the filtered mix in 0.1 M acetic acid.

#### Linearity and limit of quantification

The linearity was evaluated with linear regression on a 4-point standard curve from 4 to 100 ng L^−1^ for each sampling site in water and from 3 to 30 ng mg^−1^ in cyanobacterial filaments. Quantification was done with standard curves in each matrix for the analyte based on the peak area versus the corresponding concentration.

#### Precision and accuracy

The precision was evaluated on triplicate analysis of matrix blank from each site, spiked with 10 μL in-house reference material. The accuracy was evaluated on triplicate analyses, prepared as precision quality control, and fortified with noncertified standard at 10 ng mL^−1^. The accuracy was subtracted from the precision to obtain a value that was expected to represent the noncertified standard at 10 ng mL^−1^.

#### Recovery

The recovery was evaluated in an intraday experiment for each lake with spiked samples of matrix blank with standard and with treated tap water spiked with in-house reference material. The spiked calibration curve in surface river water and the treated tap water spiked with in-house reference material were compared with standard curve in solution. In addition, spiked field samples were compared with standard in solution. The recovery yield was calculated as follows:

$$ \mathrm{Recovery}=\frac{\mathrm{QC}\ \mathrm{spiked}\ \mathrm{before}\ \mathrm{extraction}}{\mathrm{Standard}\ \mathrm{in}\ \mathrm{solution}} $$

$$ \mathrm{Recovery}\ \mathrm{in}\ \mathrm{each}\ \mathrm{sample}=\frac{\mathrm{Sample}\ \mathrm{spiked}\ \mathrm{before}\ \mathrm{extraction}}{\mathrm{Standard}\ \mathrm{in}\ \mathrm{solution}} $$

### LWTX-1 release under laboratory conditions

A preliminary study was conducted to determine the release and degradation rates of LWTX-1 from a *M. wollei* mat kept under laboratory conditions. Cyanobacteria collected from Lake Saint-Pierre were brought to the laboratory and maintained in an aquarium filled with 2 L of treated tap water (5-μm-activated charcoal filter and UV irradiation, devoid of toxins, as verified by initial blank measurements) for 105 days at room temperature and low light intensity (≈ 10 μmol m^−2^ s^−1^). A 100-mL sample from overlying water was taken periodically with a graduated pipet and transferred into a 100-mL polypropylene bottle and analyzed for LWTX-1 using the same extraction procedure as water samples (see the “LWTX-1 extraction” section and the “Identification and quantification of LWTX-1 using LC-QqQMS” section).

### Statistical analyses

Relationships between LWTX-1 concentrations and physical, chemical, and biological variables were examined using linear regressions and Pearson r correlation coefficient (Excel). The decaying function of toxins released by the cyanobacterial mat kept under laboratory conditions was modeled using an exponential regression (Excel).

## Results

### Validation of LWTX-1 extraction method in water and filaments

Validation of the LWTX-1 method for cyanobacterial filaments was carried out previously (Lajeunesse et al. 2012). In the present study, linearity, precision, accuracy, recovery, and lower limit of quantification were evaluated to validate the extraction method developed for LWTX-1 in water. As explained in the “Method validation” section, two different matrix blanks were used to account for the different matrix effects of each tributary. St. Lawrence River surface water originating from Lake Ontario was used for Lake Saint-Louis samples and Yamaska River surface water was used for Lake Saint-Pierre samples. As expected, the matrix effect differed among water sources, with waters originating from Lake Ontario showing the smallest matrix effect.

Linearity was evaluated with linear regression in each matrix blank and coefficients of determination (*R*^2^) equalled 0.999 for Lake Saint-Louis and 0.997 for Lake Saint-Pierre in water. Lower limit of quantification (LLOQ) was evaluated for accuracy for each lake, that is, 103.9% for Lake Saint-Louis and 106.4% for Lake Saint-Pierre in water.

Precision was evaluated with in-house reference material spiked in each matrix blank. First, to evaluate the in-house reference material, a series of dilutions quantified on a standard curve in solution was carried out. The LWTX-1 concentration of in-house reference material equaled 57.6 ± 2.7 ng mg^−1^. Then, precision evaluation varies with collection site, yielding more variations in Lake Saint-Louis (27%) than in Lake Saint-Pierre (2%). Accuracy was assessed as the difference between accuracy results (matrix blank spiked with 10 μL in-house reference material and 10 ng mL^−1^ standard) and precision results (matrix blank spiked with 10 μL in-house reference material) in order to obtain the representation of the spiked standard in matrix with filament, as described in the “Method validation” section. Accuracy yielded similar results for both lakes, that is, 105% for Lake Saint-Louis and 89% for Lake Saint-Pierre.

The recovery success was evaluated in an intraday experiment for both sites, using spiked samples and reference material. The spiked calibration curve in each matrix blank was compared with standard curve in solution, which resulted in recovery rates of 72% for Lake Saint-Louis and 50% for Lake Saint-Pierre. Also, spiked dechlorinated water with in-house reference material was compared with standard curve in solution which yielded a recovery rate of 96%. In addition, spiked field samples were compared with standard in solution, which resulted in recovery rates of 46% in Lake Saint-Louis and 55% in Lake Saint-Pierre.

Repeated measurements of in-house reference material as additional quality control carried out during the analytical sequences of water samples closely matched the initial measurement for samples from both Lake Saint-Louis (60.47 ± 10.27 ng mg^−1^) and Lake Saint-Pierre (61.23 ± 1.45 ng mg^−1^).

### Relationship between toxin concentrations in water and filaments

**Fig. 3:**
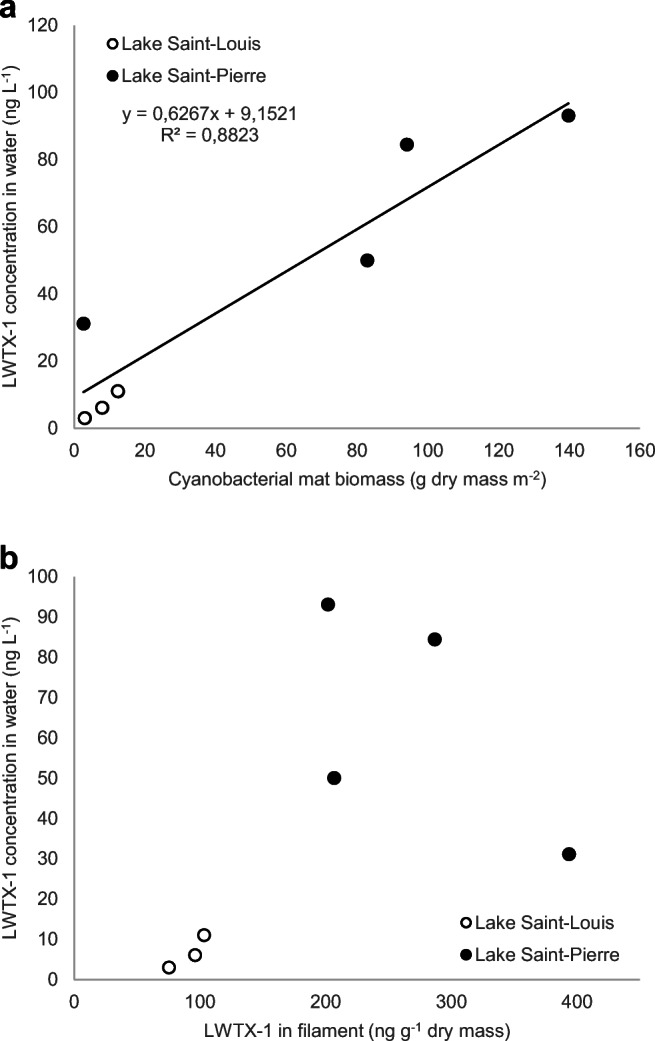
Relationship between LWTX-1 concentration in water and **a***M. wollei* biomass and **b** LWTX-1 in cyanobacterial filaments

Biomass of filament sampled at each lake ranged from 3 to 12 g DM m^2–1^ for Lake Saint-Louis and 2.6 to 139.8 g DM m^2–1^ for Lake Saint-Pierre; on average, biomass was 10-fold higher for Lake Saint-Pierre than Lake Saint-Louis. LWTX-1 was quantified in all samples and ranged from 3.01 to 11.03 ng L^−1^ in water samples and from 75.29 to 103.26 ng mg^−1^ in filament samples for Lake Saint-Louis and from 31.14 to 93.13 ng L^−1^ in water samples and from 201.99 to 393.64 ng mg^−1^ in filament samples for Lake Saint-Pierre. Higher LWTX-1 concentrations were observed in Lake Saint-Pierre, both in filaments (by 3-fold) and in the water (by 10-fold) overlying cyanobacterial mats. As hypothesized, LWTX-1 concentration in water was positively correlated (*r* = 0.94, *p* = 0.0008) with cyanobacterial mat biomass (Fig. 3a). A clear trend was observed between LWTX-1 concentration in water and toxin concentration in the cyanobacterial filaments (*r* = 0.10, n.s., Fig. 3b).

### Relationships between toxin concentrations and environmental conditions

**Fig. 4:**
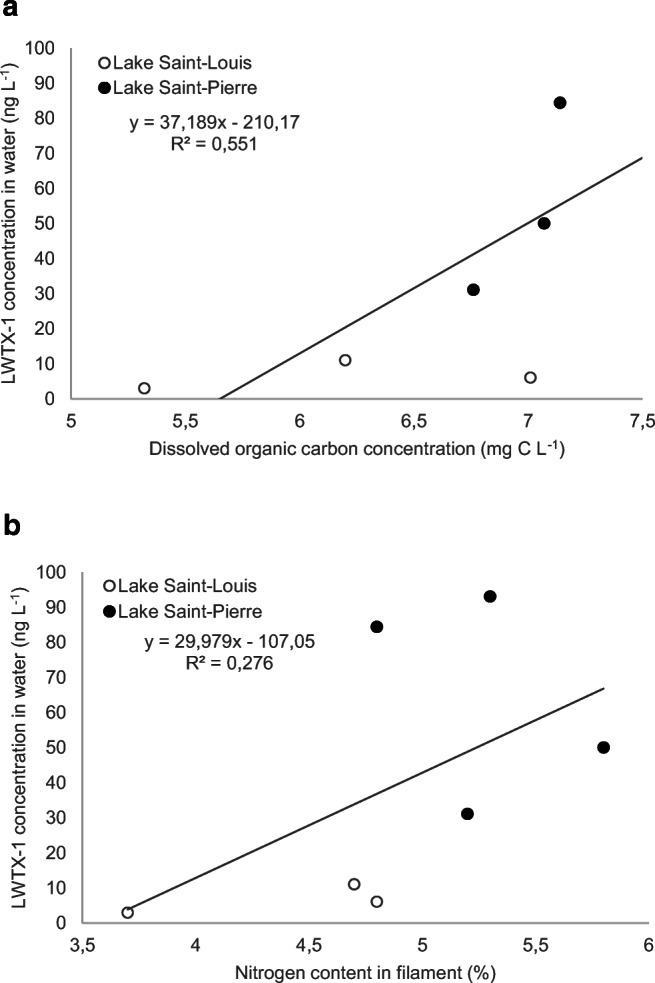
Relationships between LWTX-1 concentration in water and **a** dissolved organic carbon concentration and **b** nitrogen content in cyanobacterial filaments

Physical and chemical water quality characteristics were remarkably similar between Lake Saint-Louis and Lake Saint-Pierre in August 2017 (Table 2). Cyanobacterial mats were growing in 2 m of water, with near-bottom Secchi depth owing to low (< 5 mg L^−1^) suspended matter concentrations but DOC-rich (> 6 mg C L^−1^) colored waters (> 30 Pt/Co). Both sites exhibited moderate enrichment by phosphorus (TP < 20 μg P L^−1^) and nitrogen (TN < 600 μg N L^−1^), but low (< 200 μg N L^−1^) nitrite-nitrate concentrations. Data combined for both study sites revealed that LWTX-1 concentration in water was positively correlated with concentration of dissolved organic carbon (*r* = 0.74, *p* = 0.03, Fig. 4a) and weakly with % nitrogen content in the cyanobacterial filaments (*r* = 0.52, *p* = 0.11, Fig. 4b).

**Table 2:** Comparison of physical and chemical characteristics (mean ± SD) measured at the time of cyanobacterial mat sampling in Lake Saint-Louis (*N* = 3, 15 August 2017) and Lake Saint-Pierre (*N* = 4) (28 August 2017)

	Lake Saint-Louis	Lake Saint-Pierre
Sample depth (m)	2.03 ± 0.25	1.90 ± 0.34
Conductivity (μS cm^−1^)	200.6 ± 46.6	241.7 ± 12.7
Suspended matter (mg L^−1^)	4.0 ± 1.7	1.5 ± 0.6
Color (Pt/Co)	34.7 ± 11.7	32.0 ± 3.6
Secchi depth (m)	1.9 ± 0.1	1.8 ± 0.1
Dissolved organic carbon (mg C L^−1^)	6.2 ± 0.9	7.1 ± 0.3
Total phosphorus (μg P L^−1^)	20 ± 5	17 ± 3
Total dissolved phosphorus (μg P L^−1^)	13 ± 1	11 ± 3
Total nitrogen (μg N L^−1^)	570 ± 26	583 ± 120
Total dissolved nitrogen (μg N L^−1^)	500 ± 17	533 ± 113
Nitrites-nitrates (μg N L^−1^)	144 ± 21	166 ± 104
Ammonium (μg N L^−1^)	5 ± 6	1.5 ± 0

### Toxin release and degradation rate

**Fig. 5:**
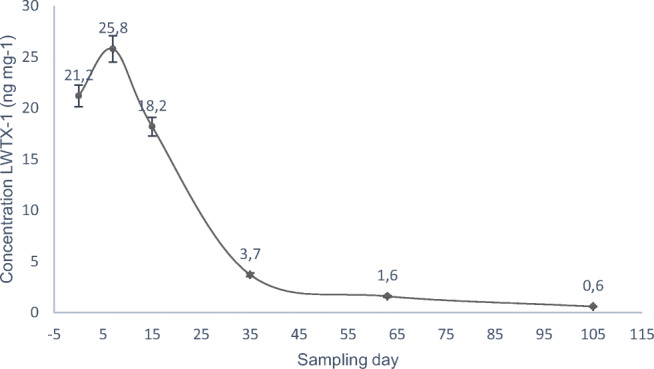
Rate of LWTX-1 release and degradation from a *M. wollei* mat maintained under controlled laboratory conditions over a 105-d period

Assays of toxin release and degradation were conducted by measuring LWTX-1 concentrations in water overlying a cyanobacterial mat kept under controlled laboratory conditions (Fig. 5). In the first week, an increase up to 26 ng mL^−1^ was observed and then concentrations dropped to near zero (0.49 mg mL^−1^) on day 105, following a typical decaying curve. This indicated that toxins were mostly released in the first week and then degraded over a period of almost 100 days. Over the same period, the color of the cyanobacterial mat slowly faded away and whitened over time. From this simple experiment, the half-life value of LWTX-1 was estimated at roughly 17 days at room temperature. Hiskia et al. (2014) reported that some transformation pathways may contribute to water detoxification from cyanotoxins: temperature and pH-dependent decomposition, biological degradation, photolysis and photosensitized degradation.

## Discussion

Our study took advantage of the wide range of *M. wollei* biomass sampled in 2017 in lakes Saint-Louis and Saint-Pierre (2.6–140 g DM m^−2^) to assess the importance of toxin release from the cyanobacterial mats into the overlying waters. Earlier records in the St. Lawrence have shown a large level of variability of *M. wollei* biomass on a spatial (within and among lakes) and a temporal (seasonally and among years) bases (Hudon et al. 2014, 2016).

The large variations of discharge and current speed prevailing in the St. Lawrence River are in sharp contrast with the hydrological stability of lakes (Smith et al. 2019) and springs (Pinowska et al. 2007) in which *M. wollei* is often found. This element brings forward the challenge of tracing the origin and advection of water masses into which toxins have been released by a cyanobacterial mat. Furthermore, current speed will determine the distance at which toxins will be advected downstream, possibly reaching drinking water intakes or recreational areas. The wind also likely exerts an influence on the release of toxins, as was shown by the strong disrupting effect of waves on cyanobacterial mats, and eventually throwing mats ashore (Lévesque et al. 2015).

The presence of water masses of different chemical signature (Table 2) flowing side by side with little lateral mixing further highlights the question of adequately identifying the water body to use as a matrix blank for toxin extraction and analysis. Since no labeled internal standard was available for LWTX-1 analysis, several precautions were taken to ensure the quality of the results. Appropriate water blanks bearing similar physicochemical properties and without toxins were sometimes difficult to find upstream of the sampling sites. Since matrix effects have a tremendous impact on results in LC-MS/MS analyses, additional quality controls were carried out with surface water samples spiked with either standard or in-house reference materials, from which accuracy reached ≥ 89% for each lake.

LWTX-1 concentrations measured in cyanobacterial filaments in the present (2017) study (75.29–103.26 μg g^−1^ in LSL and 201.99–393.64 μg g^−1^ in LSP) were higher than those previously reported in Lake Saint-Louis (51 ± 40 μg g^−1^ 2007, 2009–2011) and in Lake Saint-Pierre (25 ± 31 μg g^−1^, 2006–2008, 2012–2013) (Hudon et al. 2016). The 10-fold increase in toxin concentration in LSP was unexpected, given that this lake previously exhibited the lowest mat biomass and toxin concentrations. Results from this study however confirmed previous observations of positive correlations between LWTX-1 in filaments with ambient DOC and a weaker correlation with the % nitrogen content in the filaments, an indicator of filaments’ health conditions (Hudon et al. 2016).

Our field results show that LWTX-1 toxin occurs in significant amounts in the water above *Microseira wollei* mats, ranging from 3.01 to 11.03 ng L^−1^ in Lake Saint-Louis and from 31.14 to 93.13 ng L^−1^ in Lake Saint-Pierre. Cyanobacterial mat biomass was positively correlated with LWTX-1 concentrations in the water overlying the mats. Surprisingly, weak correlation was found between toxin concentration in filaments and water. This could be due to many factors, such as current speed, the physiological status of mats, and sampling position above the biomass mat relative to the river current. In contrast with results from the present study, healthy (planktonic) cyanobacterial populations blooming in the field were reported to produce little extracellular toxin (Chorus and Bartram 1999). For planktonic blooms, cell-bound concentrations were several orders of magnitude higher than toxins released in the water during bloom breakdown, owing to high dilution rate into ambient water (Jones and Orr 1994).

The conditions under which our laboratory experiment was conducted were coherent with the previously documented ability of *M. wollei* to survive under low light intensity (22–50 μmol m^−2^ s^−1^) (Pinowska et al. 2007). Mats collected in the field were growing under low light intensity, as shown by the depth of collection (2 m) which was just below the Secchi depth, as previously observed in the St. Lawrence (Lévesque et al. 2012, 2017b). These observations support the general tendency for *M. wollei* to grow under low light intensity, in brown, DOC-rich waters with low inorganic nitrogen (Lévesque et al. 2012, 2017a). In addition, the sharp release of LWTX-1 within the first 7 days under laboratory conditions and the persistence of the toxin for over 100 days raise concerns about its occurrence in the raw waters of drinking water filtration plants. Release of cylindrospermopsin by benthic cyanobacteria contaminated drinking water in Australian reservoirs (Gregor et al. 2007; Zamyadi et al. 2012; Gaget et al. 2017; Lorenzi et al. 2018; Almuhtaram et al. 2018), but such occurrence has been more frequently documented with planktonic cyanobacteria (Gregor et al. 2007; Zamyadi et al. 2012; Almuhtaram et al. 2018).

## Conclusion

This study provided a strong support to our hypothesis that *Microseira wollei* released cyanotoxins into the overlying waters in proportion with the biomass of mats of cyanobacterial filaments lying on the bottom. Accurate measurements of released toxins were made possible by the development of validation methods correcting for complex matrix effects resulting from the presence of variable/high DOC concentrations. LWTX-1 concentrations in the water overlying cyanobacterial mats were also correlated with ambient DOC and nitrogen content of the filaments, in agreement with earlier findings. Maintenance of mats under controlled laboratory conditions showed that the rate of LWTX-1 release was highest within the first week of sampling and followed a typical decaying curve over the next 100 days. These results emphasize the need for efficient monitoring of *M. wollei* proliferation in natural waters, to ensure environmental and public health and safety.
